# Supplementary material for: AGAMEMNON: an Accurate metaGenomics And MEtatranscriptoMics quaNtificatiON analysis suite
Source: Genome Biol. 2022 Jan 31;23:39. doi: 10.1186/s13059-022-02610-4 (PMC8802518; doi:10.1186/s13059-022-02610-4)
Supplement: Supplementary file 1 — Additional file 1: Supplementary figures S1 – S11. Figures demonstrating benchmark results between the methods compared throughout the study. AGAMEMNON results using 16 publicly available RNA-Seq tissue samples from the ENCODE consortium. [file 13059_2022_2610_MOESM1_ESM.pdf]

AGAMEMNON: an Accurate metaGenomics And MEtatranscriptoMics quaNtificatiON analysis suite

Giorgos Skoufos[1, 2, †, *], Fatemeh Almodaresi[3, †], Mohsen Zakeri[3], Joseph N Paulson[4], Rob Patro[3], Artemis G Hatzigeorgiou[1, 2, 5, #, *] & Ioannis S Vlachos[6, 7, #, *]

Additional file 1

Figures

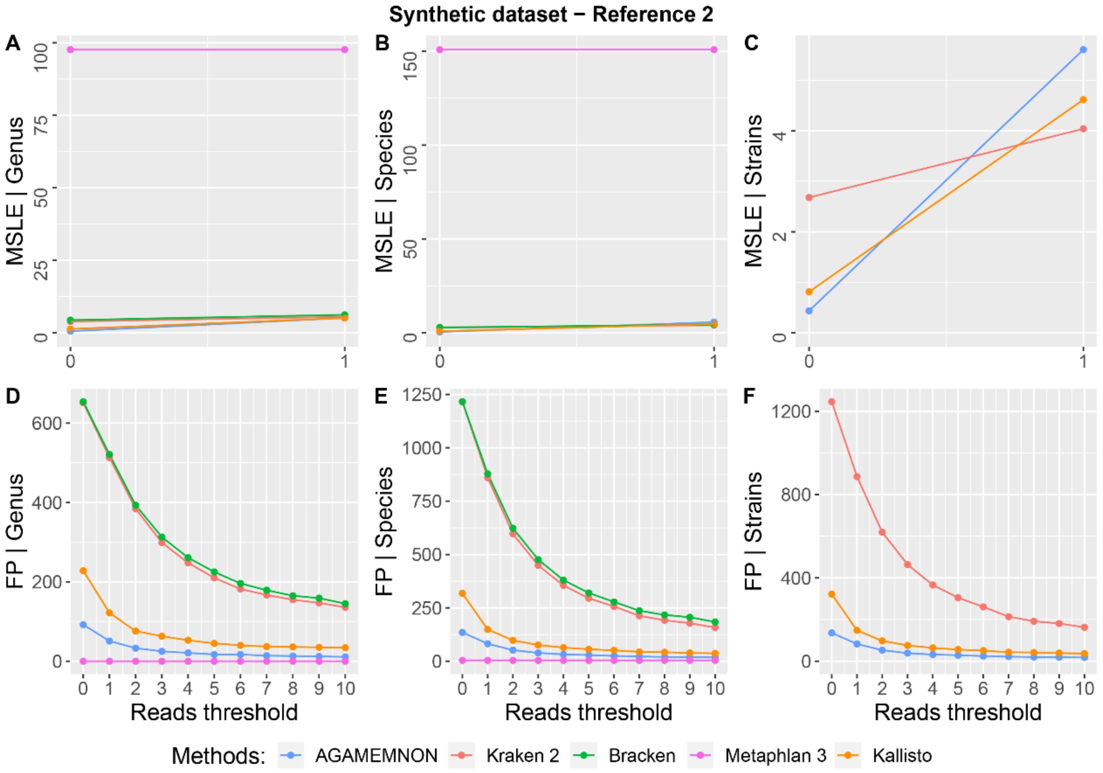

Figure S1: *The Mean Squared Log Error (MSLE) and the number of false positive taxa (FP) between true and estimated read counts at the levels of Genus, Species and Strain using the synthetic dataset and reference 2. We measured MSLE (a) using unfiltered results (0 x axis tick) and (b) by removing all instances where the true and estimated counts were both zero (1 x axis tick). False positive taxa were counted at all read thresholds between 0 and 10. At the read threshold of 0 reads (unfiltered results), all taxa were counted, even those with just 1 assigned read. At the read threshold of 1 read, we counted the taxa with > 1 assigned read and so on. Bracken and Metaphlan 3 produce results up to the species level and thus they were not included in the strain-level comparisons.*

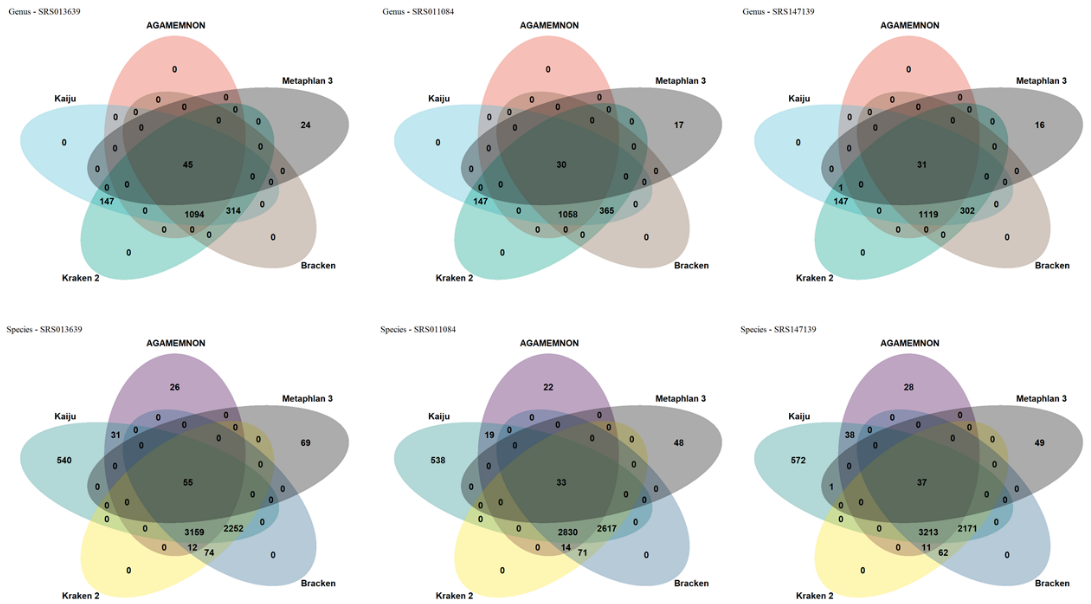

Figure S2: *Venn diagrams demonstrating the commonly & distinctly identified taxa between Metaphlan 3, Bracken, Kraken 2, Kaiju and AGAMEMNON at the levels of genus and species.*

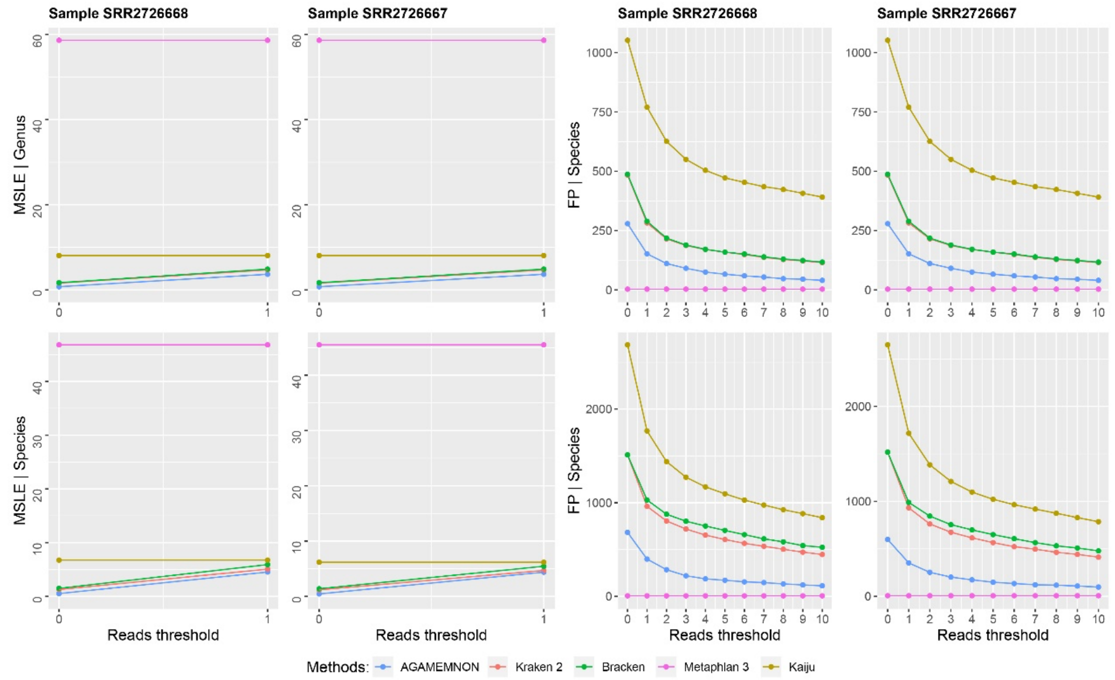

Figure S3: *The Mean Squared Log Error (MSLE) and the number of false positive taxa (FP) between true and estimated read counts at the levels of Genus and Species using reference 3 and samples from Marcus B. Jones et al., PNAS, 2015. We measured MSLE (a) using unfiltered results (0 x axis tick) and (b) by removing all instances where the true and estimated counts were both zero (1 x axis tick). False positive taxa were counted at all read thresholds between 0 and 10. At the read threshold of 0 reads (unfiltered results), all taxa were counted, even those with just 1 assigned read. At the read threshold of 1 read, we counted the taxa with > 1 assigned read and so on. In this comparison, all species present in the datasets are also present in the reference.*

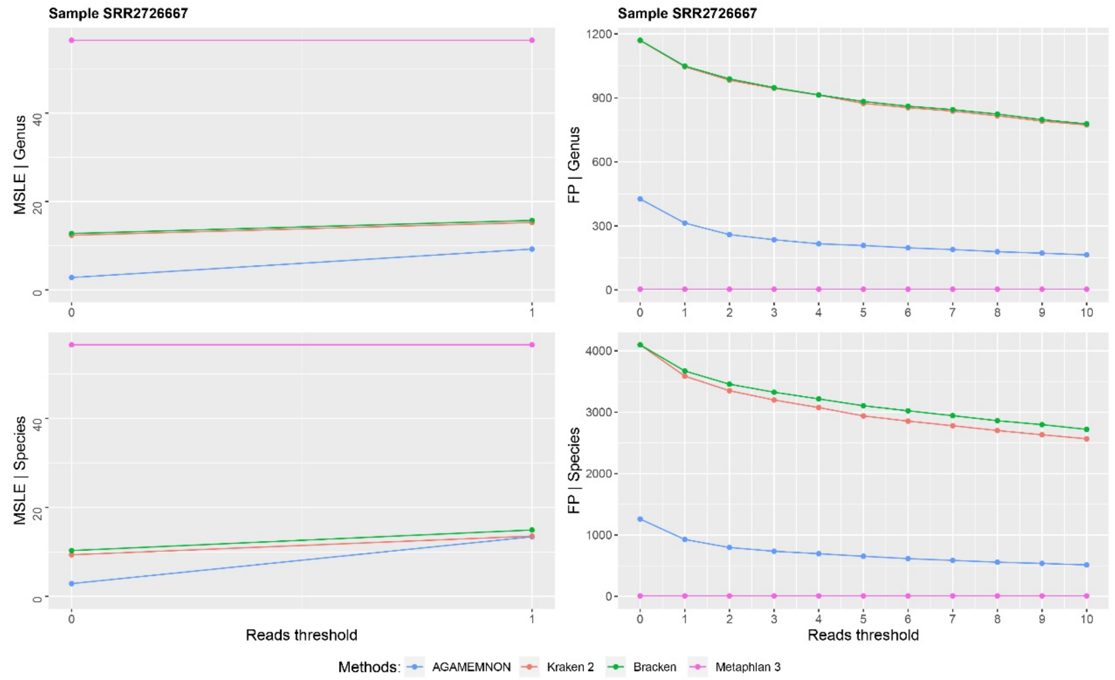

Figure S4: *The Mean Squared Log Error (MSLE) and the number of false positive taxa (FP) between true and estimated read counts at the levels of Genus and Species using reference 4 and samples from Marcus B. Jones et al., PNAS, 2015. We measured MSLE (a) using unfiltered results (0 x axis tick) and (b) by removing all instances where the true and estimated counts were both zero (1 x axis tick). False positive taxa were counted at all read thresholds between 0 and 10. At the read threshold of 0 reads (unfiltered results), all taxa were counted, even those with just 1 assigned read. At the read threshold of 1 read, we counted the taxa with > 1 assigned read and so on. Kaiju was not included in this test since its pre-set index includes all sequences of the dataset and cannot be excluded to render it comparable with the other methods. In this comparison, 9 out of the 20 species present in the dataset are missing from the reference.*

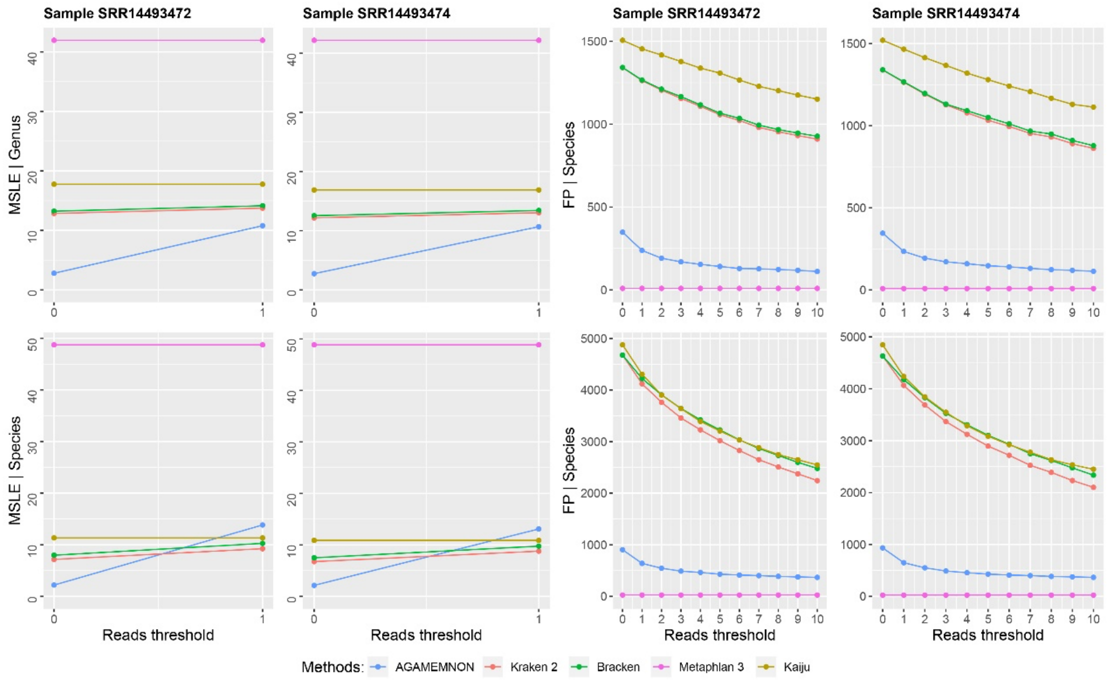

Figure S5: *The Mean Squared Log Error (MSLE) and the number of false positive taxa (FP) between true and estimated read counts at the levels of Genus and Species using reference 3 and samples from Wenyi Xu et al., frontiers in Microbiology, 2021. We measured MSLE (a) using unfiltered results (0 x axis tick) and (b) by removing all instances where the true and estimated counts were both zero (1 x axis tick). False positive taxa were counted at all read thresholds between 0 and 10. At the read threshold of 0 reads (unfiltered results), all taxa were counted, even those with just 1 assigned read. At the read threshold of 1 read, we counted the taxa with > 1 assigned read and so on. In this comparison, 16 out of the 69 species present in the dataset are missing from the reference.*

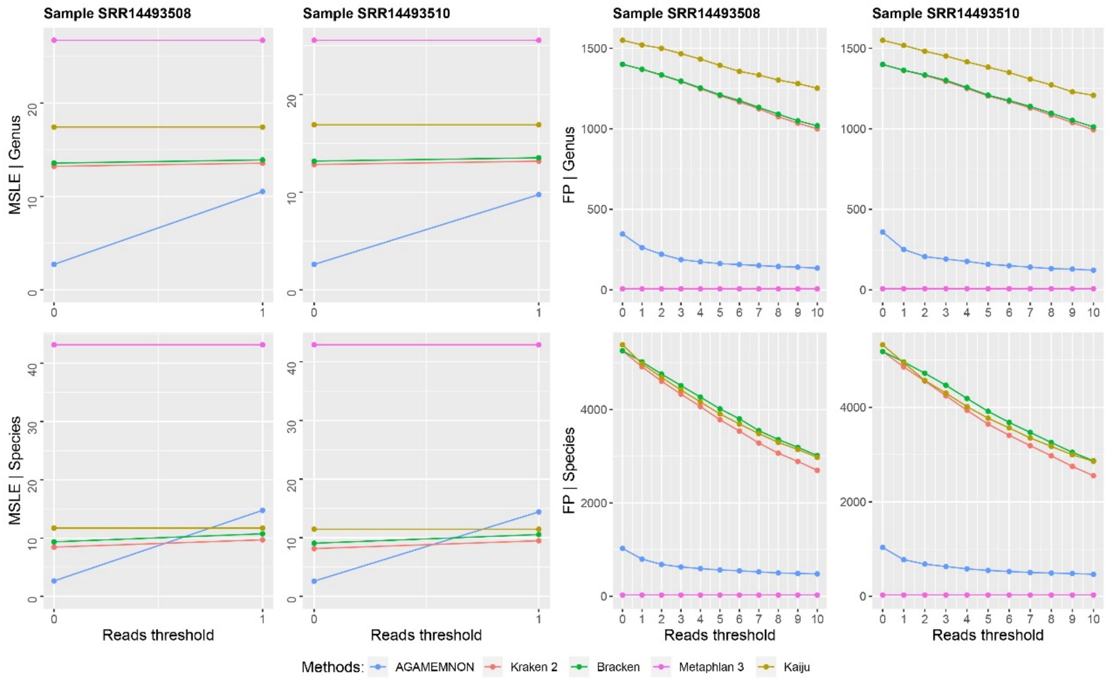

Figure S6: *The Mean Squared Log Error (MSLE) and the number of false positive taxa (FP) between true and estimated read counts at the levels of Genus and Species using reference 3 and samples from Wenyi Xu et al., frontiers in Microbiology, 2021. We measured MSLE (a) using unfiltered results (0 x axis tick) and (b) by removing all instances where the true and estimated counts were both zero (1 x axis tick). False positive taxa were counted at all read thresholds between 0 and 10. At the read threshold of 0 reads (unfiltered results), all taxa were counted, even those with just 1 assigned read. At the read threshold of 1 read, we counted the taxa with > 1 assigned read and so on. In this comparison, 19 out of the 62 species present in the dataset are missing from the reference.*

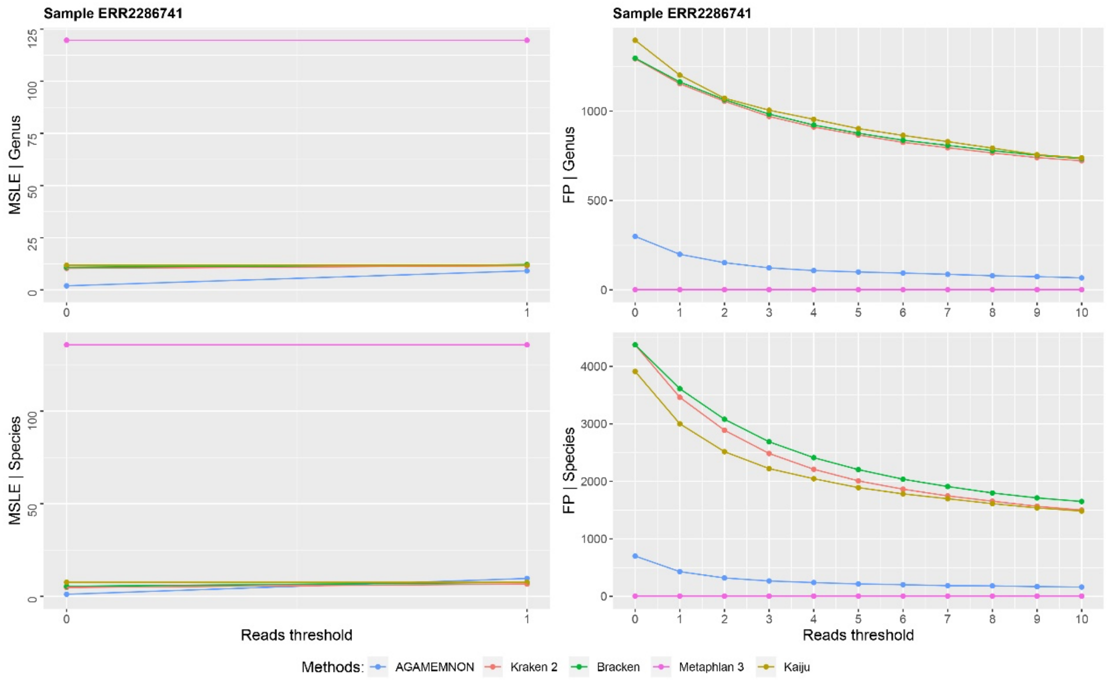

Figure S7: *The Mean Squared Log Error (MSLE) and the number of false positive taxa (FP) between true and estimated read counts at the levels of Genus and Species using reference 3 and the sample from Aaron M. Walsh et al., Microbiome, 2018. We measured MSLE (a) using unfiltered results (0 x axis tick) and (b) by removing all instances where the true and estimated counts were both zero (1 x axis tick). False positive taxa were counted at all read thresholds between 0 and 10. At the read threshold of 0 reads (unfiltered results), all taxa were counted, even those with just 1 assigned read. At the read threshold of 1 read, we counted the taxa with > 1 assigned read and so on. In this comparison, 2 out of the 13 species present in the dataset are missing from the reference.*

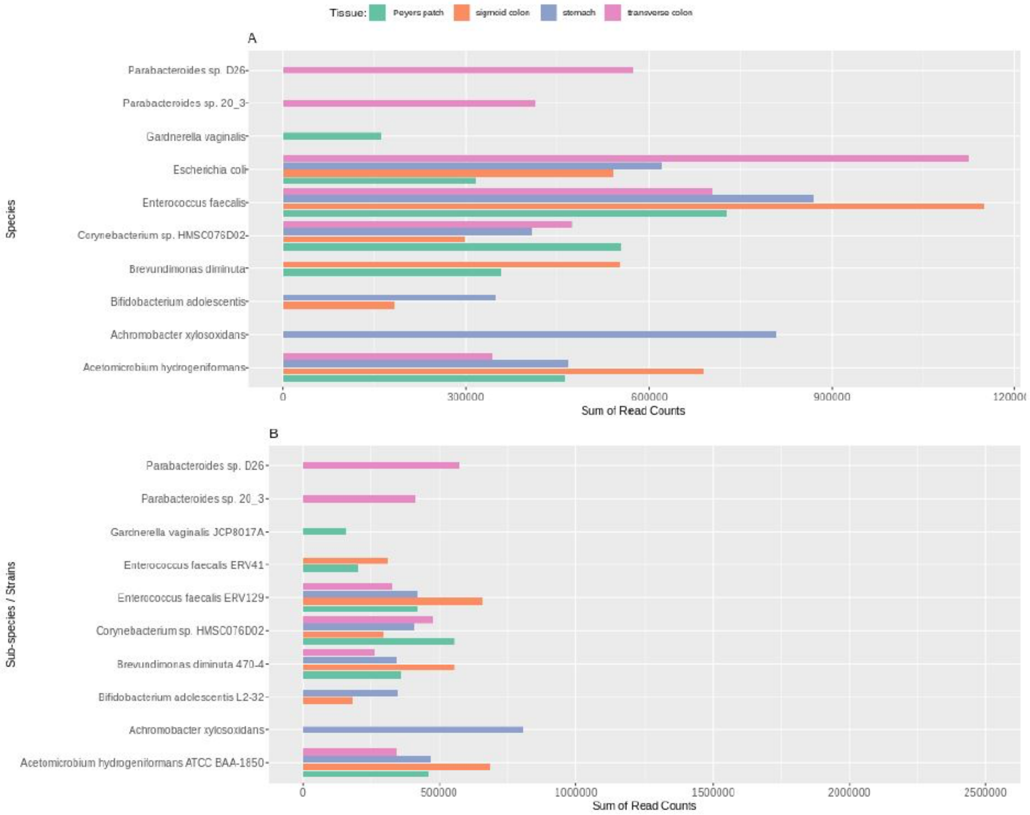

Figure S8: *Results of the ENCODE datasets using AGAMEMNON. (A) The six most abundant species across all 16 samples in terms of sum of read counts per tissue (Peyer's Patch, Sigmoid Colon, Stomach, Transverse Colon). (B) The six most abundant sub-species/strains across all 16 samples in terms of sum of read counts per tissue. Most of the identified species are known to be present in the human gut. Gardnerella Vaginalis is a rather surprising finding, which following literature evaluation it has also been reported in Zhou et al. (Front Nutr 2021) and Gopalakrishnan et al. (Science, 2018). However, since the ground truth in these datasets is not known it is not possible to evaluate further the accuracy of these quantifications.*

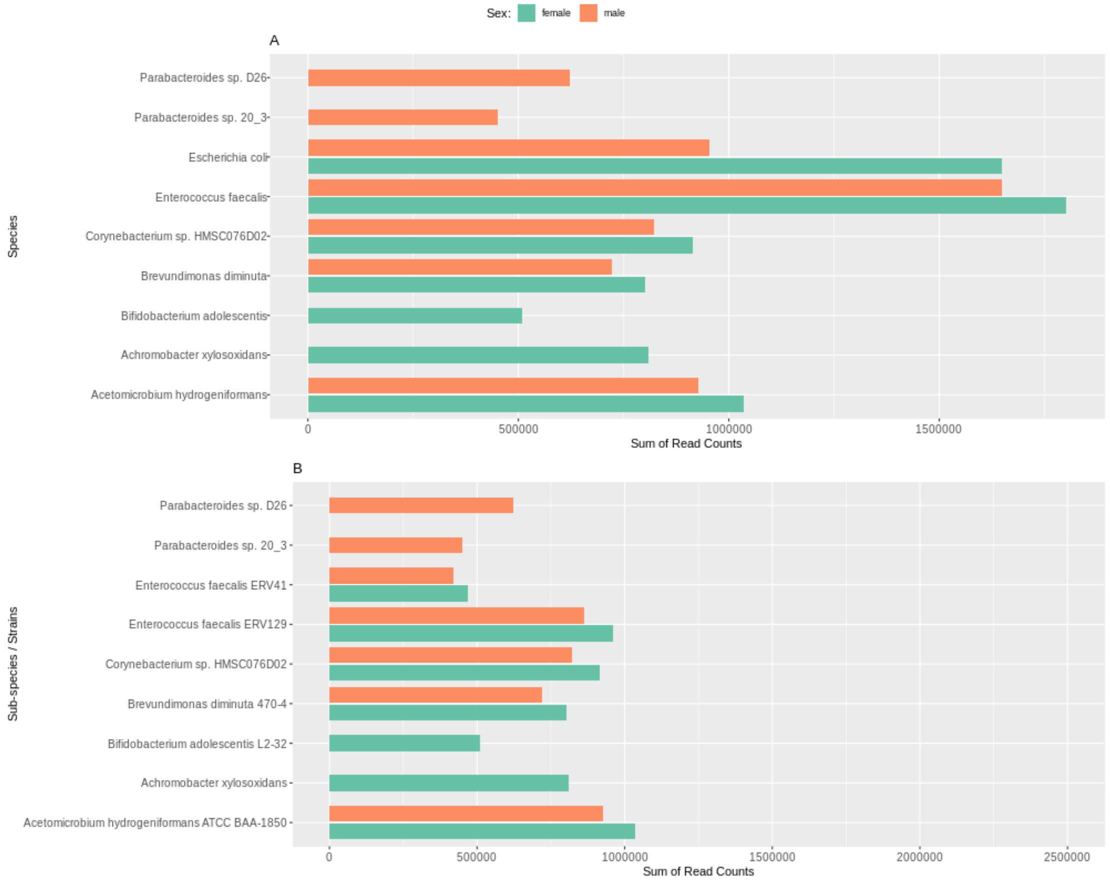

Figure S9: *Results of the ENCODE datasets using AGAMEMNON. (A) The six most abundant species across all 16 samples in terms of sum of read counts per gender. (B) The six most abundant sub-species/strains across all 16 samples in terms of sum of read counts per gender.*

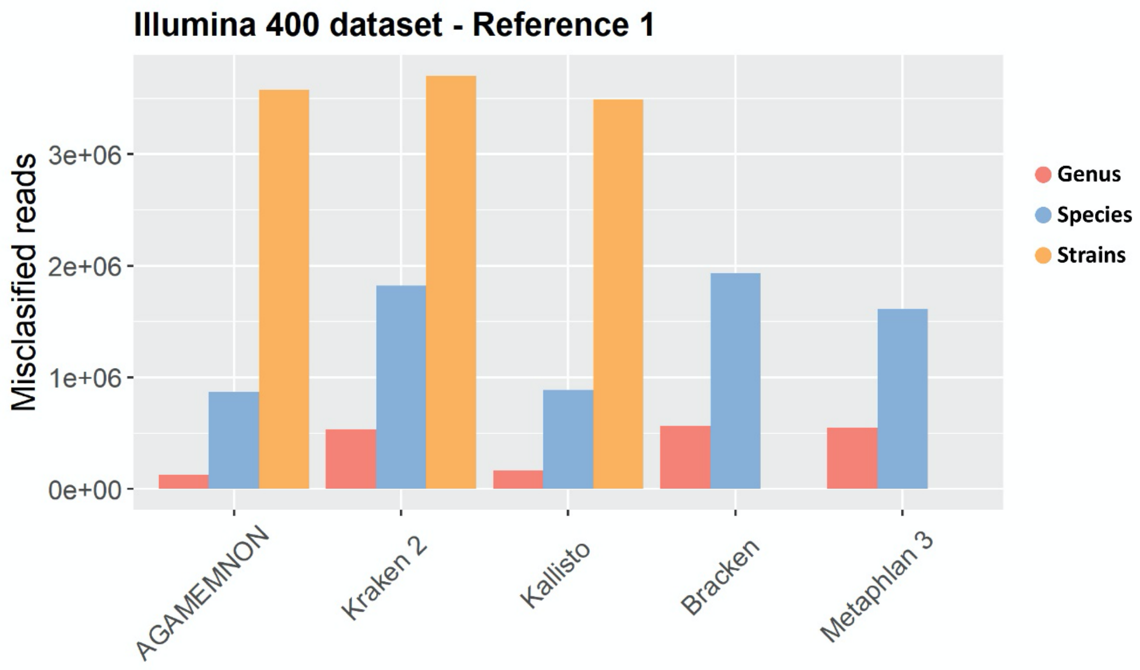

Figure S10: *Number of misclassified reads at the genus, species and strain levels (Illumina 400, Reference 1).*

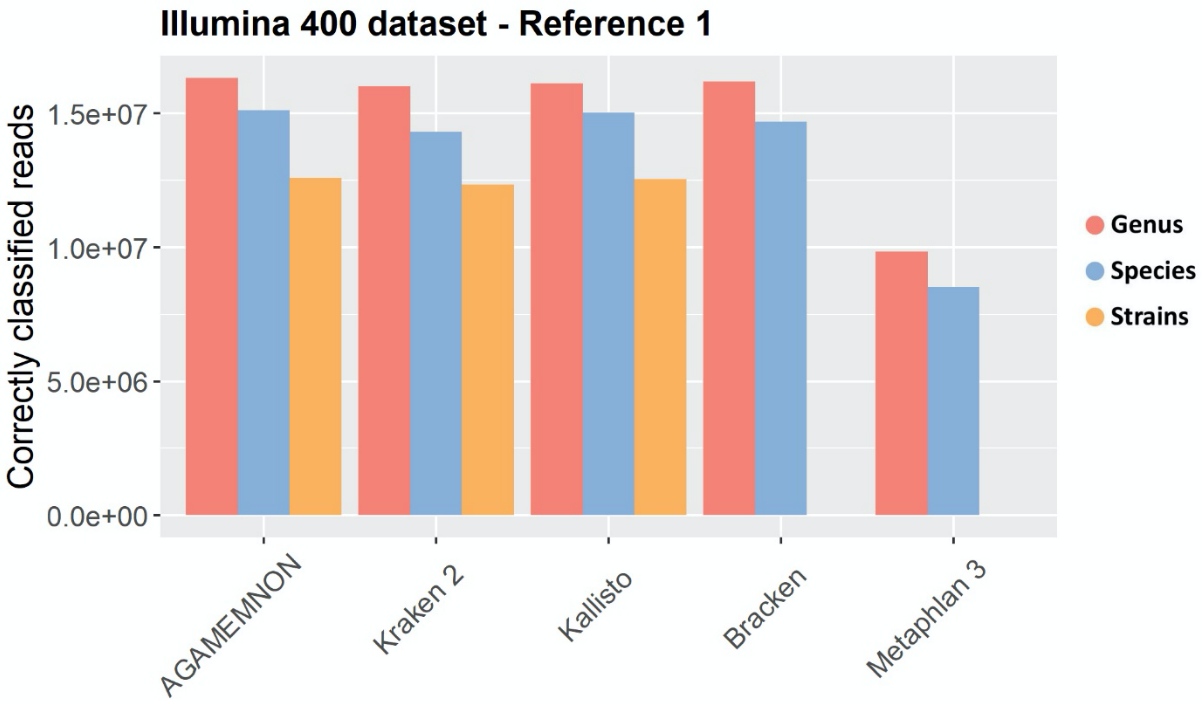

Figure S11: *Number of correctly classified reads at the genus, species and strain levels (Illumina 400, Reference 1).*
