# Supplementary material for: AGAMEMNON: an Accurate metaGenomics And MEtatranscriptoMics quaNtificatiON analysis suite
Source: Genome Biol. 2022 Jan 31;23:39. doi: 10.1186/s13059-022-02610-4 (PMC8802518; doi:10.1186/s13059-022-02610-4)
Supplement: Supplementary file 2 — Additional file 2. Demonstration of the first use-case scenario using AGAMEMNON and employing visualizations and differential abundance analyses using data from the integrated Human Microbiome Project. [file 13059_2022_2610_MOESM2_ESM.pdf]

**AGAMEMNON: an Accurate metaGenomics And MEtatranscriptoMics quaNtificatiON analysis suite**

**Giorgos Skoufos[1, 2, †, *], Fatemeh Almodaresi[3, †], Mohsen Zakeri[3], Joseph N Paulson[4], Rob Patro[3], Artemis G Hatzigeorgiou[1, 2, 5, #, *] & Ioannis S Vlachos[6, 7, #, *]**

## Additional file 2

Using 47 publicly available metagenomics datasets (**Additional file 4: Supplementary Table S7: "iHMP samples"**) from the integrated Human Microbiome Project (iHMP) [1] and the Inflammatory Bowel Disease Multiomics Database (IBDMDB), we applied AGAMEMNON to (a) quantify the abundances of the identified microbial species/strains and (b) conduct downstream analyses using our differential abundance and visualization modules. All datasets are stool samples, originating from Ulcerative Colitis (UC) and healthy controls (nonIBD). We used FastQC [2] and cutadapt [3] to quality-check and pre-process the samples and then used AGAMEMNON on its metagenomics mode.

We used AGAMEMNON's human-specific reference to map the sequencing reads and identified a median of more than 800 different taxa (strains/sub-species) per sample which agrees with relevant literature on the high complexity and diversity of human gastrointestinal tract microbiota [4]. In terms of median read counts, we identified 41 highly abundant microorganisms (median read counts > 10,000). The taxa with median read counts > 50,000 are listed in **Additional file 4: Supplementary Table S15**.

We then applied our differential abundance module on the nonIBD against UC samples **(Additional file 2: Supplementary figure S12)** to search for potential differentially abundant (DA) microorganisms between the two conditions. Even though AGAMEMNON offers six different models for differential expression/abundance analyses, we chose to use the metagenomeSeq (AGAMEMNON default method), which is especially designed for differential abundance analyses [5]. Our analyses revealed 13 differentially abundant (FDR < 0.05) microbial strains/sub-species (**Additional file 4: Supplementary Table S10: "DA microbes"**). Interestingly, the species Enterococcus faecium which is among the differentially abundant species, has been shown to promote ulcerative

colitis in mouse in a recently published study [6]. In our findings, Enterococcus faecium is more abundant in the UC patients with an FDR of 0.02, a logFC of 3.07 and its present in 100% of the UC samples and 73.9% of the healthy controls.

**Table S15:** TaxIDs and their corresponding median of read counts across all 47 samples. We only present the microorganisms with median of read counts > 50000.

| TaxID | Median of Read Counts |
|---|---|
| 469593 | 313861.13 |
| 457394 | 245562.55 |
| 997891 | 221562.33 |
| 411483 | 98634.00 |
| 818 | 96459.57 |
| 411485 | 83813.78 |
| 748224 | 74326.21 |
| 411479 | 68613.90 |
| 457393 | 53179.11 |
| 820 | 51201.23 |

Using AGAMEMNON's R/Shiny application and its visualization/exploratory modules, we made a series of plots both in the strains/species identified as DA and the rest of the results presented in **Additional file 2: Supplementary Figures S12, S13, S14 and S15**.

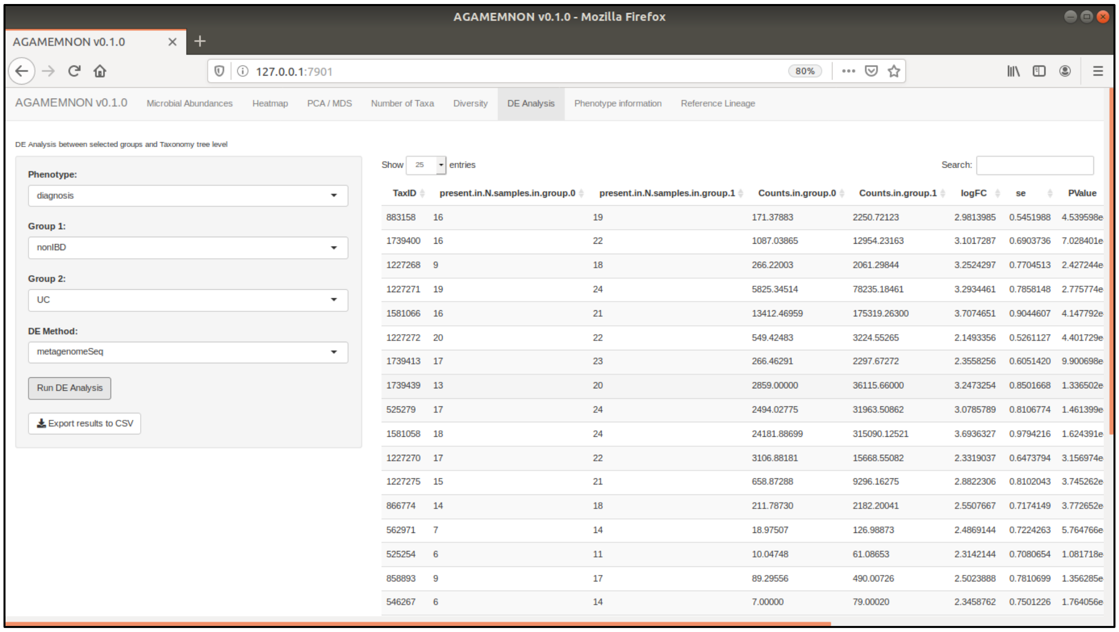


**Figure S12:** Screenshot of AGAMEMNON's R/Shiny application. On the left, there is a menu from which the user can choose the phenotypic characteristic, the groups and the method by which the DE/DA analyses will be conducted. After the execution, an interactive data table will appear on the right, containing the results of the analyses.

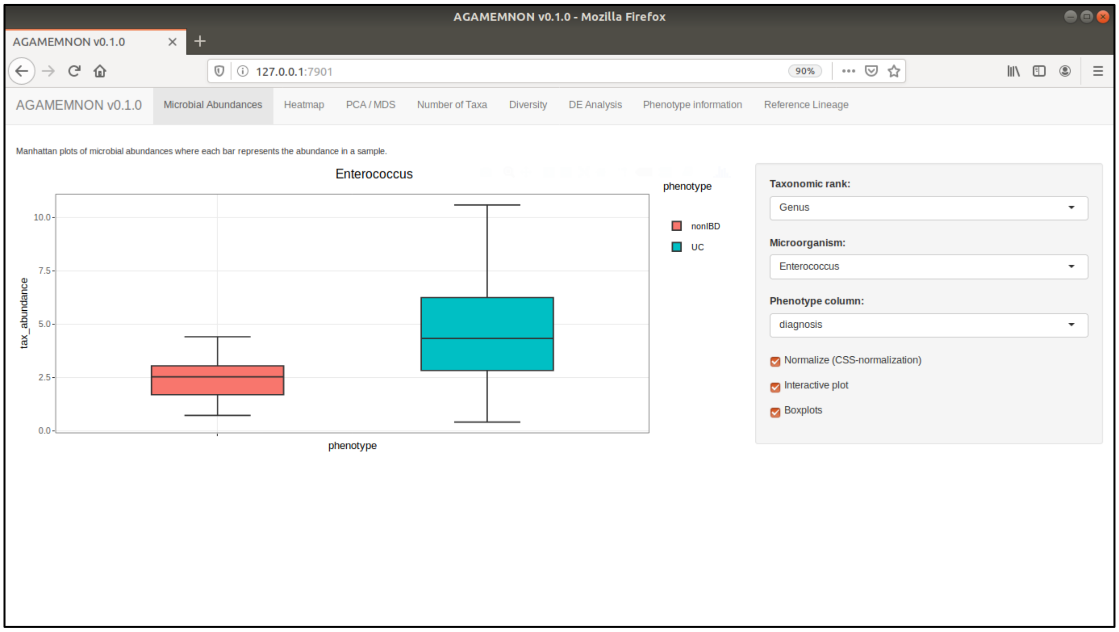


**Figure S13:** Boxplots that represents the abundance of the genus *Enterococcus* in the UC and nonIBD samples respectively.

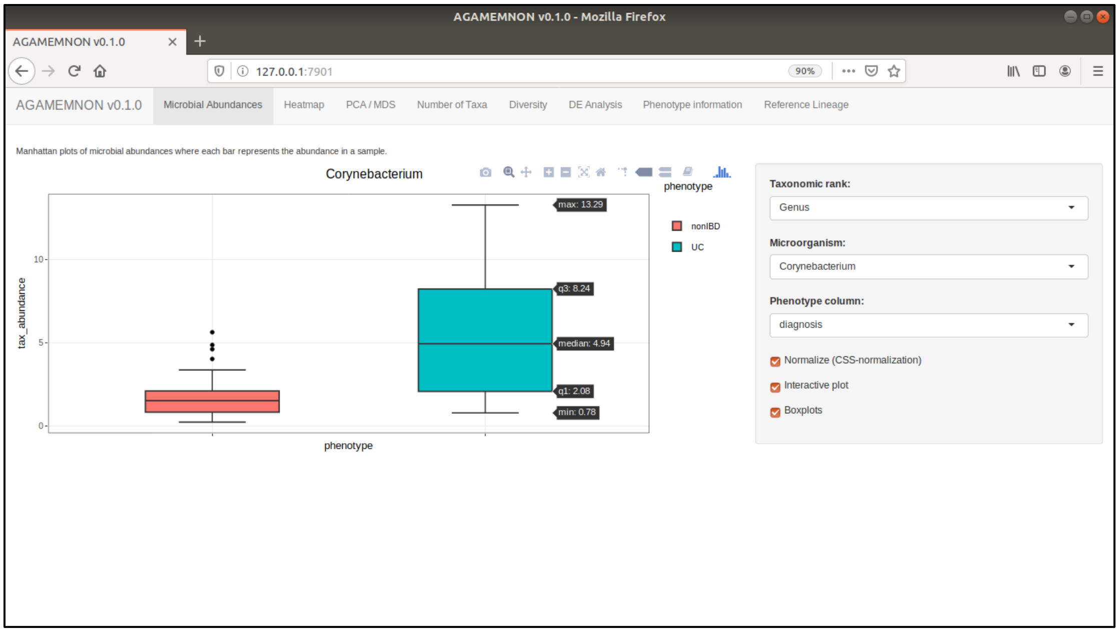


**Figure S14:** Boxplots that represents the abundance of the genus *Corynebacterium* in the UC and nonIBD samples respectively. Some *Corynebacterium* strains, identified as DA.

As shown in figures S13 and S14, we chose the Taxonomic rank, Microorganism, Phenotypic characteristic and type of plot on the right and the results, automatically appeared on the left of the R/Shiny application. This is a fast/easy way to visualize some of the microorganisms identified as DE/DA or any other quantified microorganism of interest.

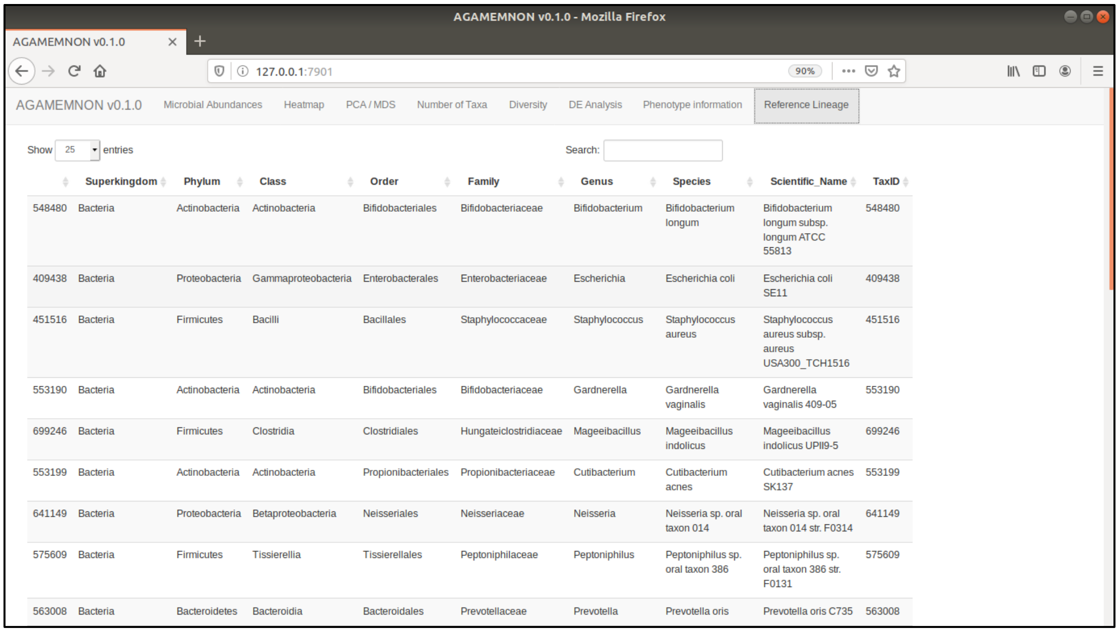


**Figure S15:** Interactive data table of the full lineage of the microbial genomes used as a reference on the Human Microbiome Project samples analyses. In the data table presented here, the user can search for specific microorganisms using their corresponding TaxID or any other taxonomic level and get their full lineage.

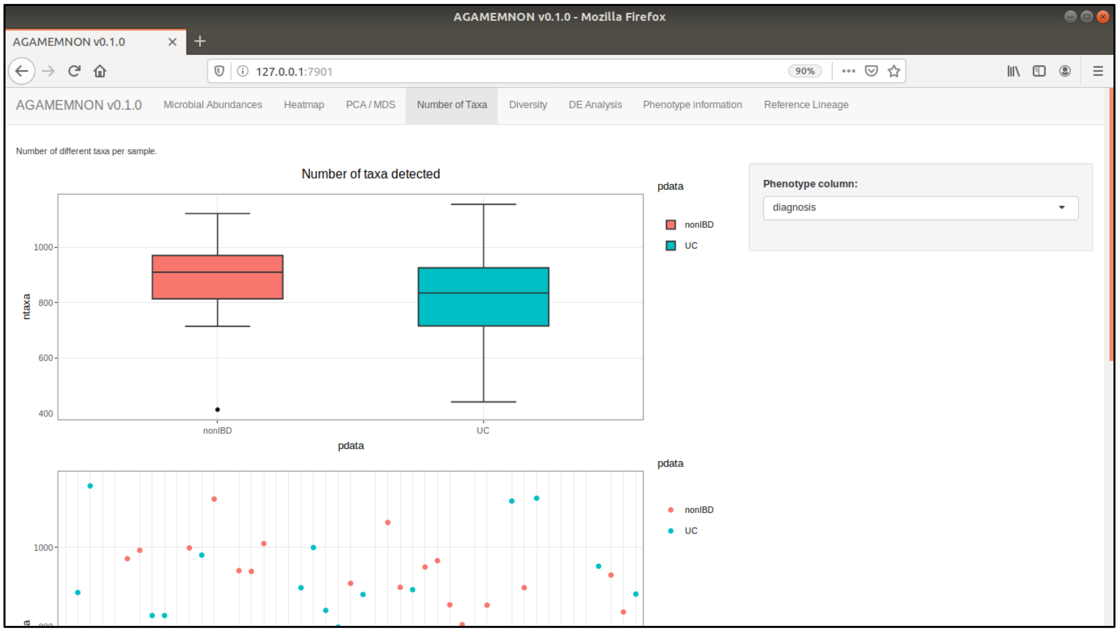


**Figure 1.5:** Boxplots and a Manhattan plot of the number of different taxa (strains/sub-species) identified in the samples. On the right of AGAMEMNON's R/Shiny application, we chose the phenotypic characteristic by which we wanted to group the

samples. We selected to visualize the number of taxa detected in relation their diagnosis (i.e., Ulcerative colitis patients or Healthy controls)

In conclusion, we started our analyses with 47 publicly available raw datasets, we quantified the abundances of the microbial genomes identified in the samples, we then conducted a differential abundance analysis between a selected condition, identified a number of DA microorganisms and created a series of plots to explore our results further.

The above, is a small use-case scenario conducted A-to-Z using solely AGAMEMNON.

# References


1. Integrative HMPRNC: **The Integrative Human Microbiome Project: dynamic analysis of microbiome-host omics profiles during periods of human health and disease.** *Cell host & microbe* 2014, **16:**276-289.

2. Andrews S: **FastQC: a quality control tool for high throughput sequence data.** 2010.

3. Martin M: **Cutadapt removes adapter sequences from high-throughput sequencing reads.** *2011* 2011, **17:**3.

4. Lozupone, C.A., Stombaugh, J.I., Gordon, J.I., Jansson, J.K. & Knight, R: **Diversity, stability and resilience of the human gut microbiota.** Nature 489, 220-230 (2012).

5. Paulson JN, Stine OC, Bravo HC, Pop M: **Differential abundance analysis for microbial marker-gene surveys.** *Nature Methods* 2013, **10:**1200.

6. Seishima, J. et al.: **Gut-derived Enterococcus faecium from ulcerative colitis patients promotes colitis in a genetically susceptible mouse host.** Genome Biology 20, 252 (2019).
