# Supplementary material for: AGAMEMNON: an Accurate metaGenomics And MEtatranscriptoMics quaNtificatiON analysis suite
Source: Genome Biol. 2022 Jan 31;23:39. doi: 10.1186/s13059-022-02610-4 (PMC8802518; doi:10.1186/s13059-022-02610-4)
Supplement: Supplementary file 3 — Additional file 3. Demonstration of the first use-case scenario using AGAMEMNON and employing visualizations and differential abundance analyses using data from the Feng et al. [file 13059_2022_2610_MOESM3_ESM.pdf]

AGAMEMNON: an Accurate metaGenomics And MEtatranscriptoMics quaNtificatiON analysis suite

Giorgos Skoufos[1, 2, †, *], Fatemeh Almodaresi[3, †], Mohsen Zakeri[3], Joseph N Paulson[4], Rob Patro[3], Artemis G Hatzigeorgiou[1, 2, 5, #, *] & Ioannis S Vlachos[6, 7, #, *]

Additional file 3

Using 24 publicly available metagenomics datasets (**Additional file 4: Supplementary Table S9: "BENCHMARK-Real samples")** from Feng *et al.* [1], we applied AGAMEMNON to (a) quantify the abundances of the identified microbial species/strains and (b) conduct downstream analyses using our differential abundance and visualization modules. All datasets are stool samples, originating from colon carcinoma (8 samples) and colon adenoma (8 samples) patients, as well as healthy controls (8 samples). We used FastQC [2] and cutadapt [3] to quality-check and pre-process the samples and then used AGAMEMNON on its metagenomics mode.

We used AGAMEMNON's REF-3 reference to map the sequencing reads and identified a median of ~1100 different taxa (strains/sub-species > 5 assigned reads) per sample. In terms of median read counts, we identified 48 highly abundant microorganisms (median read counts > 10,000). The taxa with median read counts > 50,000 are listed in **Additional file 4: Supplementary Table S16**.

We then applied our differential abundance module and conducted pairwise comparisons between all three conditions (i.e., carcinoma, adenoma, controls) to search for potential differentially abundant (DA) microorganisms. We chose to use metagenomeSeq (AGAMEMNON default method), which is especially designed for differential abundance analyses [4]. In the carcinoma versus control comparison, our analysis revealed 11 differentially abundant (FDR < 0.05) microbial strains/sub-species (**Additional file 4: Supplementary Table S11 "DA microbes-car-con"**). Interestingly, 3 out of the 11 DA strains/sub-species, belong to the species Fusobacterium nucleatum whose role in colorectal carcinogenesis is well studied [5 - 7]. In our findings, Fusobacterium nucleatum is more abundant in the carcinoma patients. FDR, logFC and % presence

of the three Fusobacterium nucleatum strains/sub-species are listed in **Additional file 4: Supplementary Table S17**.

Table S16: *TaxIDs and their corresponding median of read counts across all 24 samples. We only present the microorganisms with median of read counts > 50000.*

TaxID	Median of Read Counts
853	500388.20
2479767	332565.00
649756	330082.00
39488	198444.50
410072	191573.50
239935	157330.04
74426	126349.40
411483	123469.00
585394	97479.30
536231	84029.55
820	80564.06
1679	71587.04
1550024	67498.45
2093857	58931.70
411903	52645.85
216816	50909.56
357276	50503.15

Table S17: *TaxID, FDR, logFC and % presence of the three Fusobacterium nucleatum strains/sub-species found to be DA between the carcinoma and healthy control samples*

TaxID	FDR	logFC	% Presence in carcinoma	% Presence in controls
155615	0	3.55	100	12.5
525283	2.38E-10	3.10	100	0
469602	2.68E-05	2.62	100	12.5

Using AGAMEMNON's R/Shiny application and its visualization/exploratory modules, we also made a series of plots both in the strains/species identified as DA and the rest of the results presented in **Additional file 3: Supplementary Figures S17-S19**.

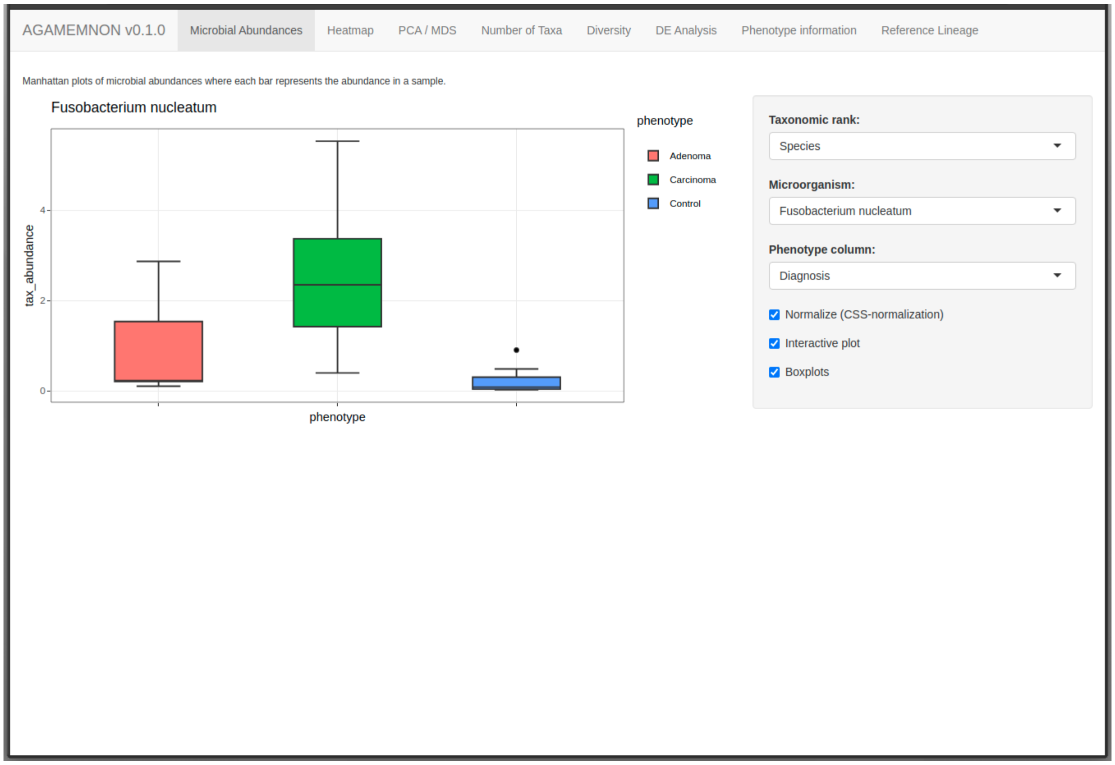

Figure S17: *Boxplots that represents the abundance of the species Fusobacterium nucleatum in the adenoma (red), carcinoma (green) and control (blue) samples respectively.*

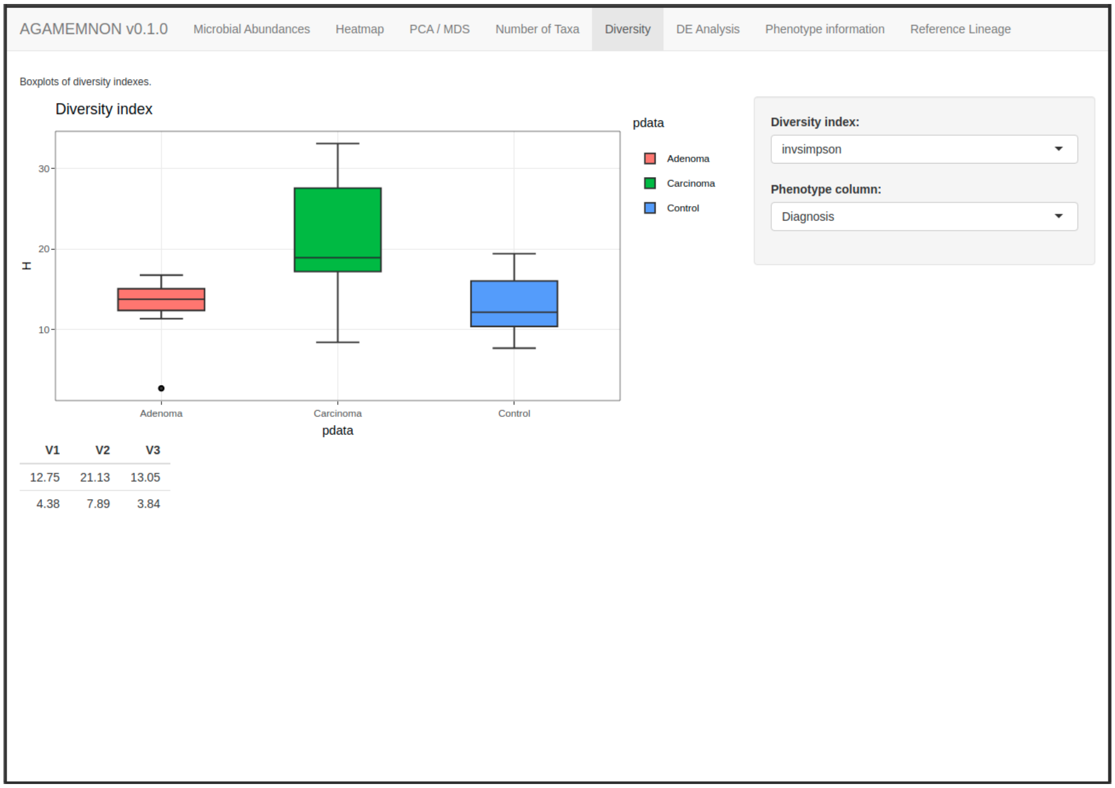

Figure S18: *Boxplots representing the per group InvSimpson diversity index.*

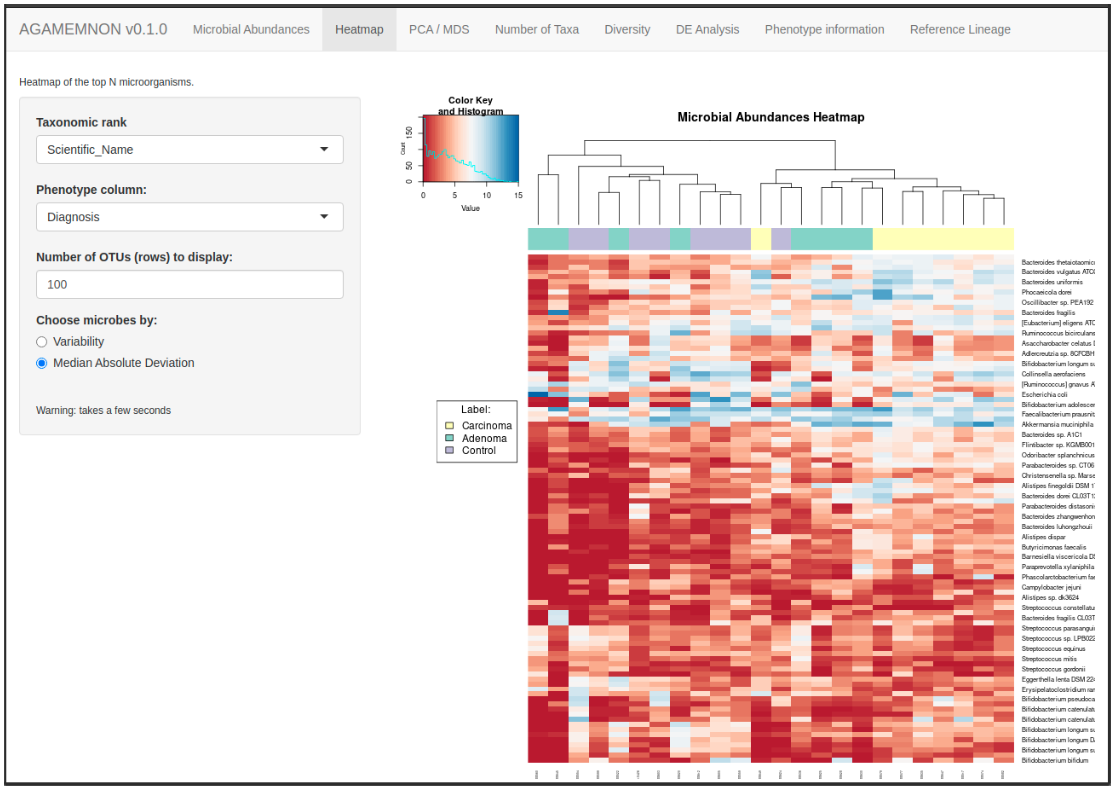

Figure S19: *Microbial abundances heatmap using all 24 samples and the 100 most abundant strains. Except for one carcinoma sample, the rest are closely related (yellow top right) while the adenoma and control samples are mixed.*

In the carcinoma versus adenoma and adenoma versus control comparisons, our analyses revealed 10 and 3 DA microorganisms respectively (**Additional file 4: Supplementary Tables S12: "DA microbes-car-ade" and S13: "DA microbes-ade-con"**).

The aforementioned scenario is another small use-case scenario conducted A-to-Z using AGAMEMNON.

References

1. Feng Q, Liang S, Jia H, Stadlmayr A, Tang L, Lan Z, Zhang D, Xia H, Xu X, Jie Z, et al: **Gut microbiome development along the colorectal adenoma–carcinoma sequence.** *Nature Communications* 2015, **6:**6528.

2. Andrews S: **FastQC: a quality control tool for high throughput sequence data.** 2010.

3. Martin M: **Cutadapt removes adapter sequences from high-throughput sequencing reads.** *2011* 2011, **17:**3.

4. Paulson JN, Stine OC, Bravo HC, Pop M: **Differential abundance analysis for microbial marker-gene surveys.** *Nature Methods* 2013, **10:**1200.

5. Rubinstein MR, Baik JE, Lagana SM, Han RP, Raab WJ, Sahoo D, Dalerba P, Wang TC, Han YW: **Fusobacterium nucleatum promotes colorectal cancer by inducing Wnt/β-catenin modulator Annexin A1.** *EMBO reports* 2019, **20:**e47638.

6. Shang F-M, Liu H-L: **Fusobacterium nucleatum and colorectal cancer: A review.** *World journal of gastrointestinal oncology* 2018, **10:**71-81.

7. Sun C-H, Li B-B, Wang B, Zhao J, Zhang X-Y, Li T-T, Li W-B, Tang D, Qiu M-J, Wang X-C, et al: **The role of Fusobacterium nucleatum in colorectal cancer: from carcinogenesis to clinical management.** *Chronic diseases and translational medicine* 2019, **5:**178-187.
